# Phthalate Exposure and Allergy in the U.S. Population: Results from NHANES 2005–2006

**DOI:** 10.1289/ehp.1206211

**Published:** 2013-06-25

**Authors:** Jane A. Hoppin, Renee Jaramillo, Stephanie J. London, Randi J. Bertelsen, Päivi M. Salo, Dale P. Sandler, Darryl C. Zeldin

**Affiliations:** 1 Epidemiology Branch, National Institute of Environmental Health Sciences (NIEHS), National Institutes of Health (NIH), Department of Heath and Human Services (DHHS), Research Triangle Park, North Carolina, USA; 2 SRA International, Durham, North Carolina, USA; 3 Division of Environmental Medicine, Norwegian Institute of Public Health, Oslo, Norway; 4 Laboratory of Respiratory Biology, NIEHS, NIH, DHHS, Research Triangle Park, North Carolina, USA

## Abstract

Background: Environmental exposures to phthalates, particularly high-molecular-weight (HMW) phthalates, are suspected to contribute to allergy.

Objective: We assessed whether phthalate metabolites are associated with allergic symptoms and sensitization in a large nationally representative sample.

Methods: We used data on urinary phthalate metabolites and allergic symptoms (hay fever, rhinitis, allergy, wheeze, asthma) and sensitization from participants ≥ 6 years of age in the National Health and Nutrition Examination Survey (NHANES) 2005–2006. Allergen sensitization was defined as a positive response to at least one of 19 specific IgE antigens (≥ 0.35 kU/L). Odds ratios (ORs) per one log_10_ unit change in phthalate concentration were estimated using logistic regression adjusting for age, race, body mass index, gender, creatinine, and cotinine. Separate analyses were conducted for children (6–17 years of age) and adults.

Results: The HMW phthalate metabolite monobenzyl phthalate (MBzP) was the only metabolite positively associated with current allergic symptoms in adults (wheeze, asthma, hay fever, and rhinitis). Mono-(3-carboxypropyl) phthalate and the sum of diethylhexyl phthalate metabolites (both representing HMW phthalate exposures) were positively associated with allergic sensitization in adults. Conversely, in children, HMW phthalate metabolites were inversely associated with asthma and hay fever. Of the low-molecular-weight phthalate metabolites, monoethyl phthalate was inversely associated with allergic sensitization in adults (OR = 0.79; 95% CI: 0.70, 0.90).

Conclusion: In this cross-sectional analysis of a nationally representative sample, HMW phthalate metabolites, particularly MBzP, were positively associated with allergic symptoms and sensitization in adults, but there was no strong evidence for associations between phthalates and allergy in children 6–17 years of age.

Citation: Hoppin JA, Jaramillo R, London SJ, Bertelsen RJ, Salo PM, Sandler DP, Zeldin DC. 2013. Phthalate exposure and allergy in the U.S. population: results from NHANES 2005–2006. Environ Health Perspect 121:1129–1134; http://dx.doi.org/10.1289/ehp.1206211 [Online 25 June 2013].

## Introduction

Phthalates are common industrial chemicals used in cosmetics, personal care products, plastics, and building materials. Exposures are frequent, but the contributions of specific sources are poorly characterized. Phthalates represent a broad chemical class that includes both low-molecular-weight (LMW) compounds such as diethyl phthalate (DEP) and relatively high-molecular-weight (HMW) compounds such as diethylhexyl phthalate (DEHP) and butylbenzyl phthalate (BBzP). LMW phthalates are most commonly found in cosmetics and personal care products, whereas HMW phthalates are most associated with plastics, particularly polyvinyl chloride (PVC) building materials (Buckley et al. 2012; Carlstedt et al. 2012; Hauser and Calafat 2005). Results from dietary intervention studies suggest that food packaging is the primary source of human exposure to DEHP, but not BBzP (Koch et al. 2013; Rudel et al. 2011). BBzP exposure is associated with PVC flooring and other building materials in the home (Carlstedt et al. 2012).

Phthalates and other plasticizing chemicals have been associated with wheeze, allergies, and asthma among children (Bornehag and Nanberg 2010; Bornehag et al. 2004; Choi et al. 2010; Hsu et al. 2011; Just et al. 2012a, 2012b; Kolarik et al. 2008; Larsson et al. 2007) and adults (Jaakkola and Knight 2008; Jaakkola et al. 2006). Recent studies have measured phthalate levels in urine or dust, although earlier studies suggested a role for phthalates due to the presence of plastic materials in the home (Jaakkola and Knight 2008; Larsson et al. 2007), exposure to PVC plastics in an occupational setting (Jaakkola and Knight 2008) or use of synthetic bedding (Ponsonby et al. 2003). As the evidence for a potential role for phthalates in respiratory and allergic outcomes has increased, there is greater interest in HMW phthalates such as DEHP and BBzP, with evidence both from human and animal studies (Dearman et al. 2009; Deutschle et al. 2008; Jaakkola and Knight 2008; Koike et al. 2009; Larsen et al. 2007; Nishioka et al. 2012). These HMW phthalates alter immune responses in animal and *in vitro* models (Koike et al. 2009; Larsen et al. 2007). In addition, other studies suggest that DEHP and other plasticizers may act as adjuvants to enhance the allergic response (Kimber and Dearman 2010).

Recent reports have stressed the need to understand the potential allergic health effects of phthalates (Dodson et al. 2012; Hulin et al. 2012; Kwak et al. 2009). Although some evidence suggests a role of phthalates in the etiology of allergic sensitization and allergic symptoms, there is a paucity of population-based data, particularly among adults. To address this, we evaluated the association of specific phthalate metabolites with measures of allergic symptoms and sensitization in a representative sample of the U.S. population, the National Health and Nutrition Examination Survey (NHANES) 2005–2006.

## Methods

We used publicly available data from NHANES 2005–2006 [Centers for Disease Control and Prevention (CDC 2012)] to evaluate the association of phthalates and allergy. NHANES 2005–2006 collected detailed data on allergic symptoms and sensitization, so both questionnaire and biochemical measures of allergy are available for all NHANES participants > 1 year of age (*n* = 8,338). At the time of recruitment, all study participants provided informed consent. All data were anonymized prior to becoming publicly available. Urinary phthalate concentrations were measured in a random sample of participants ≥ 6 years of age (*n* = 2,548). Our analysis is limited to the 2,325 individuals who had complete information on allergy, urinary phthalate concentrations, and model covariates.

We assessed both self-reported current allergic symptoms and allergic sensitization as measured by specific IgE (sIgE). Information on current allergic symptoms was obtained from self-administered questionnaires completed at the NHANES clinic visit. Subjects < 16 years of age were interviewed with a proxy respondent, usually a parent, responsible for completing the interview. The questionnaire asked about six allergic conditions (asthma, wheeze, hay fever, allergy, itchy rash, and rhinitis) in the past year.

Serum samples were analyzed for allergen-specific IgEs using the Pharmacia Diagnostics ImmunoCAP 1000 System (Kalamazoo, MI, USA). A total of 19 allergen-specific IgEs *(Dermatophagoides farinae, Dermatophagoides pteronyssinus*, cat, dog, cockroach, *Alternaria alternata,* peanut, egg white, cow’s milk, ragweed, rye grass, bermuda grass, oak, birch, shrimp, *Aspergillus fumigatus*, Russian thistle, mouse, and rat) were assessed. Individuals who tested positive (≥ 0.35 kU/L) to at least one allergen were considered allergen sensitized (sIgE positive). Information on sensitization to specific allergens from NHANES 2005–2006 has been published elsewhere (Salo et al. 2011).

Fifteen phthalate metabolites were measured in spot urine samples using high performance liquid chromatography–electrospray ionization–tandem mass spectrometry (HPLC-ESI-MS/MS) at the National Center for Environmental Health laboratory (CDC, Atlanta, GA, USA) (CDC 2009). Four of these analytes were primary [mono-(2-ethyl)-hexyl phthalate (MEHP)] or secondary metabolites [mono-2-ethyl-5-carboxypentyl phthalate (MECPP), mono-(2-ethyl-5-hydroxyhexyl) phthalate (MEHHP), and mono(2-ethyl-5-oxohexyl) phthalate (MEOHP)] of DEHP. We summed the concentrations of all four metabolites to create a summary DEHP variable (ΣDEHP) for analysis; individual DEHP metabolites were not analyzed because of their common sources and the resulting high correlation among these metabolites (77–98%). We analyzed all chemicals that were detected in ≥ 25% of the population; for values below the detection limit, we assigned a value of the limit of detection (LOD) divided by the square root of 2 (Hornung and Reed 1990).

Information on covariates was obtained either via questionnaire (e.g., demographic characteristics, smoking status) or via measurement [e.g., body mass index (BMI)]. Urinary creatinine levels were measured using the Jaffe rate reaction with a CX3 analyzer (Beckman Instruments, Brea, CA, USA).

We used logistic regression models adjusted for study design using sampling weights to estimate associations of urinary phthalates with measures of allergic sensitization and allergic symptoms. Urinary phthalate concentrations were log_10_-transformed because of nonnormality of the distribution. Models were adjusted for age (continuous), race/ethnicity (non-Hispanic white, non-Hispanic black, Mexican American, other), gender, creatinine (log_10_ transformed, continuous), BMI (categories), and cotinine (categories). Cotinine was classified as < LOD (0.015 ng/mL), low exposure (< 10 ng/mL) and high exposure (≥ 10 ng/mL). For adults, BMI was calculated as body weight in kilograms divided by height in meters squared and categorized as underweight or normal (< 25), overweight (25 to 30), or obese (≥ 30). For children, BMI was classified as the age percentile underweight or normal (< 85th percentile), overweight (85–95th percentile), obese (≥ 95th percentile) (Krebs et al. 2007). Similar modeling strategies have been employed for previous analyses of these outcomes in the NHANES 2005–2006 data (Salo et al. 2011). We also evaluated poverty income ratio [PIR, three categories: low (≤ 1.3), middle (1.3–3.5), and high (> 3.5) income] as a potential confounder because previous analyses have shown an association between socioeconomic status and phthalate concentrations in women (Kobrosly et al. 2012) and allergic sensitization in children in NHANES 2005–2006 (Visness et al. 2009). Adjustment for PIR did not substantially alter our odds ratio (OR) estimates. Therefore, to maximize the observations included in our models, we did not include PIR as a covariate (100 missing observations). Data for children (6–17 years of age) and adults were analyzed separately because both the covariate structure and the outcome prevalence differed between adults and children. Because phthalate concentrations and allergic sensitization rates differ by race/ethnicity, we assessed potential interaction by race/ethnicity in four categories by including three two-way interaction terms in our models and performed a likelihood ratio test (3 degrees of freedom) comparing the fit of models with and without the interaction terms to assess whether statistical interaction was present. In addition, we explored whether findings for monobenzyl phthalate (MBzP) and allergic symptoms were related to allergic sensitization by expanding our logistic regression models to four-level polytomous models (allergic sensitization + symptom, symptom without sensitization, sensitization without symptom, and no symptom + no sensitization) for each of the four symptoms (asthma, wheeze, rhinitis, hay fever). To test whether the ORs differed across the four strata, we used a contrast statement; a *p*-value for difference was the result of this contrast test. All statistical modeling was done using survey procedures in SAS, version 9.3 (SAS Institute Inc., Cary, NC, USA). A *p*-value ≤ 0.05 was considered statistically significant.

## Results

Rhinitis was the most common symptom among both children and adults (Table 1). Current hay fever was reported half as often in children (3.6%) as in adults (7.4%). Allergen sensitization was common, with 46% of children and 44% of adults being sensitized to at least one sIgE.

**Table 1:** Demographic, medical, and allergic characteristics for the adults (*n* = 1,546) and children 6–17 years of age (*n* = 779) with urinary phthalate metabolite data, NHANES 2005–2006.

Characteristic		Children		Adults	
		*n*	Weighted percent (SE)	*n*	Weighted percent (SE)
Age (years)^*a*^		779	11.9 (0.1)	1,546	45.6 (0.9)
Race/ethnicity	EGPhE	216	63.5 (3.8)	752	72.2 (3.1)
	Non-Hispanic black	223	12.3 (2.0)	372	11.6 (2.2)
	Mexican American	274	13.2 (1.7)	311	7.7 (1.0)
	Other	66	10.9 (1.9)	111	8.4 (1.3)
Gender	EGPhE	372	47.1 (2.3)	792	51.1 (1.7)
	Male	407	52.9 (2.3)	754	48.9 (1.7)
Cotinine (ng/mL)	EGPhE	169	22.0 (3.3)	286	17.9 (2.0)
	Low (≥ 0.015–10)	564	71.3 (3.4)	849	52.7 (2.0)
	High (≥ 10)	46	6.7 (0.9)	411	29.5 (1.1)
BMI^*b*^	EGPhE	505	70.7 (2.4)	494	33.2 (1.4)
	Overweight	123	16.4 (1.9)	515	32.1 (1.4)
	Obese	151	12.9 (2.1)	537	34.8 (1.7)
Current allergic conditions^*c*^	EGPhE	125	18.1 (2.6)	290	22.9 (1.0)
	Asthma	65	8.4 (1.2)	116	7.4 (0.8)
	Hay fever	23	3.6 (0.9)	88	7.4 (0.9)
	Itchy rash	43	5.2 (1.0)	118	7.8 (0.7)
	Rhinitis	188	27.6 (2.7)	498	35.4 (1.2)
	Wheeze	80	10.7 (1.6)	219	16.6 (1.3)
Allergic sensitization—any sIgE		406	46.1 (2.8)	717	44.0 (1.0)
^***a***^Weighted mean is reported for age. ^***b***^Child BMI covariate is age percentile; adult BMI covariate is actual BMI (kg/m^2^). ^***c***^Self-reported current symptoms in past 12 months; current symptoms of asthma, hay fever, or allergy were assessed only among those who reported a doctor’s diagnosis.					

Both LMW and HMW phthalates were detected in the urine of all participants (Table 2). Monoethyl phthalate (MEP) was the most commonly detected LMW phthalate metabolite. All the HMW phthalate metabolites, except mono-isononyl phthalate (MiNP), mono-cyclohexyl phthalate (MCHP), and mono-*n*-octyl phthalate (MOP) were detectable in the majority of samples. The distributions of all LMW and HMW phthalates spanned three orders of magnitude. Concentrations and distributions of phthalates were similar for children and adults (see Supplemental Material, Tables S1 and S2, respectively).

**Table 2:** Urinary phthalate metabolite concentrations (μg/L) for NHANES 2005–2006 participants.

Metabolite	LOD	> LOD (%)	GM (GSE)	Percentile				
				5th	25th	50th	75th	95th
LMW	
MiBP	Mono-isobutyl phthalate	0.3	97.1	5.19 (0.30)	0.47	2.48	5.70	11.75	31.96
MnBP	Mono-*n*-butyl phthalate	0.6	99.5	19.55 (0.81)	2.91	10.19	20.06	39.88	106.30
MEP	Monoethyl phthalate	0.5	99.6	109.24 (6.33)	11.92	38.52	101.83	288.68	1457.35
MMP	Mono-*n*-methyl phthalate	1.1	38.0	1.51 (0.07)	< LOD	< LOD	< LOD	2.52	12.46
HMW	
MBzP	Monobenzyl phthalate	0.2	98.4	8.22 (0.52)	0.68	3.72	8.73	20.45	66.64
MCOP	Mono(carboxyoctyl) phthalate	0.7	95.1	5.35 (0.36)	0.55	2.35	4.98	10.86	52.74
MCNP	Mono(carboxynonyl) phthalate	0.6	89.9	2.71 (0.11)	< LOD	1.32	2.61	5.21	17.42
MCPP	Mono-(3-carboxypropyl) phthalate	0.2	96.1	2.04 (0.10)	0.22	0.98	2.02	4.21	13.04
MCHP	Mono-cyclohexyl phthalate	0.6	2.2	0.44 (0.00)	< LOD	< LOD	< LOD	< LOD	< LOD
MiNP	Mono-isononyl phthalate	1.2	13.0	1.05 (0.02)	< LOD	< LOD	< LOD	< LOD	3.52
MOP	Mono-*n*-octyl phthalate	1.8	1.1	1.32 (0.00)	< LOD	< LOD	< LOD	< LOD	< LOD
∑DEHP	∑Diethylhexyl phtalate			85.47 (4.10)	11.36	36.23	77.28	174.02	930.24
MEHP	Mono-(2-ethyl)-hexyl phthalate	1.2	66.6	3.01 (0.13)	< LOD	< LOD	2.40	6.26	41.37
MECPP	Mono-2-ethyl-5-carboxypentyl phthalate	0.6	100.0	38.28 (1.90)	5.10	16.44	34.40	78.83	384.99
MEHHP	Mono-(2-ethyl-5-hydroxyhexyl) phthalate	0.7	99.8	25.35 (1.23)	2.85	10.51	23.33	55.02	305.09
MEOHP	Mono-(2-ethyl-5-oxohexyl) phthalate	0.7	98.9	16.14 (0.81)	1.85	6.63	14.99	35.30	184.95
Abbreviations: GM, geometric mean; GSE, geometric standard error of the mean. Below LOD fill values were determined as LOD divided by the square root of 2; imputed values were used in the calculation of GM (GSE). Restricted to participants with all covariates in logistic regression model age, race/ethnicity, gender, cotinine, BMI, creatinine.									

MBzP was the metabolite most consistently associated with allergic symptoms in adults (Table 3). It was positively associated with current asthma [OR = 1.46; 95% confidence interval (CI): 1.01, 2.11], current wheeze (OR = 1.78; 95% CI: 1.22, 2.60), current hay fever (OR = 1.68; 95% CI: 1.09, 2.59), and current rhinitis (OR = 1.24; 95% CI: 1.01, 1.52). In models adjusted for PIR, the OR for MBzP and current asthma increased (1.54; 95% CI: 0.98, 2.42) but was no longer statistically significant; no other estimates changed their statistical significance after PIR adjustment. No other HMW phthalate metabolite was significantly associated with allergic symptoms in adults. Current asthma in children was inversely associated with ΣDEHP and mono(carboxyoctyl) phthalate (MCOP), but not with MBzP or other HMW metabolites (Table 4). Individual metabolites also were inversely associated with current hay fever in children, specifically, the butyl phthalate metabolites mono-isobutyl phthalate (MiBP) and mono-*n*-butyl phthalate (MnBP), as well as mono-(3-carboxylpropyl) phthalate (MCPP), and MBzP. MEP was inversely associated with current hay fever in adults, but not children. No phthalates were associated with current itchy rash or current allergy in either children or adults (data not shown).

**Table 3:** Associations [OR (95% CI)^*a*^] between urinary phthalate metabolite concentration and current allergic symptoms in adults (*n* = 1,596), NHANES 2005–2006.

Metabolite	Current asthma (*n* = 116)	Current wheeze (*n* = 219)	Current hay fever (*n* = 88)	Current rhinitis (*n* = 498)
LMW
MiBP	1.39 (0.77, 2.50)	0.92 (0.57, 1.48)	0.93 (0.46, 1.87)	0.99 (0.76, 1.29)
MnBP	1.75 (0.67, 4.56)	1.36 (0.74, 2.53)	1.23 (0.54, 2.79)	1.34 (0.83, 2.17)
MEP	1.12 (0.80, 1.57)	1.06 (0.81, 1.39)	0.68 (0.47, 1.00)	1.03 (0.85, 1.23)
MMP	1.29 (0.70, 2.37)	1.20 (0.80, 1.79)	0.59 (0.33, 1.05)	0.91 (0.66, 1.25)
HMW
MBzP	1.46 (1.01, 2.11)	1.78 (1.22, 2.60)	1.68 (1.09, 2.59)	1.24 (1.01, 1.52)
MCOP	0.96 (0.73, 1.25)	0.83 (0.58, 1.18)	0.64 (0.37, 1.11)	0.97 (0.76, 1.25)
MCNP	0.99 (0.65, 1.49)	1.09 (0.79, 1.52)	0.66 (0.41, 1.07)	0.93 (0.59, 1.44)
MCPP	1.40 (0.78, 2.54)	1.41 (0.85, 2.34)	0.83 (0.43, 1.60)	0.98 (0.74, 1.30)
∑DEHP	1.16 (0.82, 1.64)	1.23 (0.86, 1.77)	1.09 (0.59, 2.01)	1.09 (0.86, 1.38)
All models were adjusted for age, race, gender, BMI, creatinine, and cotinine. ^***a***^ORs for 1-log_10_ increase in urinary phthalate concentration.				

**Table 4:** Associations [OR (95% CI)^*a*^] between urinary phthalate metabolite concentrations and current allergic symptoms in children 6–17 years of age (*n* = 779), NHANES 2005–2006.

Metabolite	Current asthma (*n* = 65)	Current wheeze (*n* = 80)	Current hay fever (*n* = 23)	Current rhinitis (*n* = 188)
LMW
MiBP	0.92 (0.26, 3.29)	1.08 (0.49, 2.35)	0.12 (0.04, 0.39)	0.84 (0.53, 1.33)
MnBP	0.63 (0.20, 2.02)	0.45 (0.20, 0.98)	0.07 (0.03, 0.17)	0.83 (0.46, 1.52)
MEP	0.90 (0.44, 1.85)	0.99 (0.46, 2.16)	0.58 (0.16, 2.13)	0.89 (0.65, 1.23)
MMP	1.15 (0.68, 1.95)	1.10 (0.67, 1.80)	1.01 (0.31, 3.25)	1.32 (0.80, 2.17)
HMW
MBzP	1.06 (0.33, 3.45)	0.92 (0.35, 2.37)	0.42 (0.22, 0.79)	1.02 (0.62, 1.67)
MCOP	0.74 (0.36, 1.52)	1.16 (0.65, 2.07)	0.54 (0.11, 2.56)	1.40 (0.83, 2.37)
MCNP	0.50 (0.25, 0.97)	0.81 (0.31, 2.12)	0.76 (0.13, 4.58)	1.23 (0.71, 2.13)
MCPP	0.69 (0.33, 1.43)	0.87 (0.48, 1.58)	0.12 (0.02, 0.63)	1.02 (0.65, 1.58)
∑DEHP	0.26 (0.14, 0.49)	0.58 (0.24, 1.42)	0.78 (0.18, 3.48)	1.52 (0.86, 2.66)
All models were adjusted for age, race, gender, BMI, creatinine, and cotinine. ^***a***^ORs for 1-log_10_ increase in urinary phthalate concentration.				

Phthalate metabolites, particularly those from HMW chemicals, were positively associated with allergic sensitization in adults, but not in children (Table 5). Specifically, ΣDEHP and MCPP were significantly associated with being sIgE positive. Conversely, MEP was inversely associated with allergic sensitization in adults; other LMW phthalates were not. Similar findings were observed when we analyzed allergen subgroups (e.g., indoor allergens; data not shown).

**Table 5:** ORs (95% CIs)^*a*^ for sIgE sensitization^*b*^ with urinary phthalate metabolite concentration in adults and children 6–17 years of age, NHANES 2005–2006.

Metabolite	Adults	Children
LMW
MiBP	1.32 (0.99, 1.76)	0.93 (0.51, 1.70)
MnBP	1.14 (0.74, 1.74)	1.14 (0.68, 1.93)
MEP	0.79 (0.70, 0.90)	1.24 (0.80, 1.94)
MMP	0.88 (0.65, 1.20)	0.83 (0.56, 1.23)
HMW
MBzP	1.41 (0.96, 2.06)	1.18 (0.74, 1.86)
MCOP	1.21 (0.95, 1.54)	0.69 (0.40, 1.18)
MCNP	1.23 (0.86, 1.75)	0.73 (0.44, 1.23)
MCPP	1.53 (1.12, 2.10)	0.69 (0.46, 1.03)
∑DEHP	1.41 (1.12, 1.79)	1.14 (0.79, 1.65)
All models adjusted for age, race, gender, BMI, creatinine, and cotinine. ^***a***^OR for 1 log_10_ unit change in urinary phthalate level. ^***b***^Positive for at least one of 19 allergen-specific IgEs (≥ 0.35 kU/L).		

**Figure 1:**
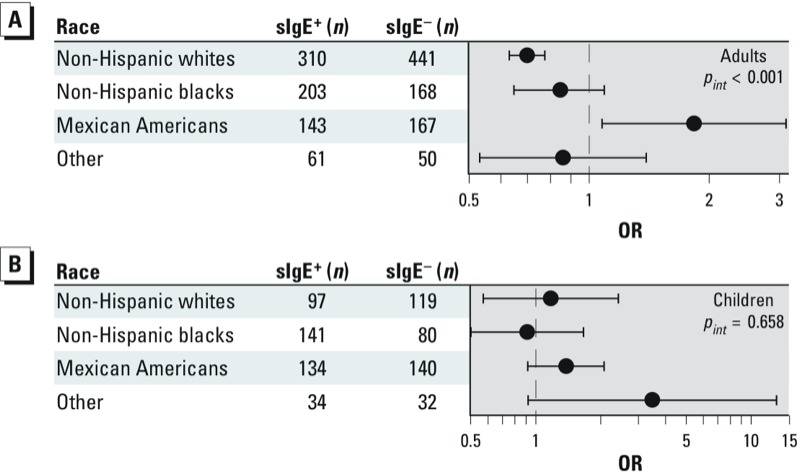
MEP and allergic sensitization by race in adults (*A*) and children 6–17 years of age (*B*).

When we evaluated potential interactions by race/ethnicity, only the interaction between MEP and sIgE sensitization in adults was statistically significant (interaction *p*-value < 0.001). As shown in Figure 1A, a log_10_ increase in MEP concentration was positively associated with sensitization in Mexican-American adults, but inversely associated with sensitization in all other adult race/ethnicity groups. Among children, the interaction by race/ethnicity was not significant and the pattern was less clear. The results suggested that in Mexican-American children, there was a positive association with MEP and sIgE (OR = 1.38; 95% CI: 0.92, 2.07; Figure 1B) and potentially an association with children of other race/ethnicity (OR = 3.72; 95% CI: 0.87, 15.87); however, the sample size was small and the CI wide, and there was no association among non-Hispanic whites and blacks.

The results from polytomous regression models of associations between MBzP and each of the four symptoms (asthma, wheeze, rhinitis, hay fever) in the presence or absence of allergic sensitization (allergic sensitization + symptom, symptom without sensitization, sensitization without symptom, and no symptom + no sensitization) suggest that adults with allergen sensitization were more likely to have wheeze (*p*_difference_ < 0.001) and asthma (*p*_difference_ = 0.011) associated with MBzP than those without sensitization or with allergic sensitization alone (Table 6). For hay fever and rhinitis there was no statistical difference in the ORs for those with and without sensitization. For children, although there was no association with MBzP with allergic sensitization or symptoms alone, when we assessed the symptoms based on allergic sensitization status, individuals with both allergic sensitization and symptoms had the highest odds of exposure. Although the individual ORs had 95% CIs that included the null value, the contrast tests for differences among the ORs indicated significant differences for wheeze based on 80 cases (*p*_difference_ = 0.047) and hay fever based on 23 cases (*p*_difference_ = 0.011).

**Table 6:** ORs (95% CIs)^*a*^ for MBzP and allergic symptoms, stratified by allergic sensitization in adults and children 6–17 years of age, NHANES 2005–2006.

Symptom/outcome	Adults			Children		
	*n*	OR (95% CI)	*p*_difference_^*b*^	*n*	OR (95% CI)	*p*_difference_^*b*^
Asthma
No sensitization, no asthma	786	1.00		353	1.00
Current asthma only	40	0.71 (0.38, 1.31)		18	0.485 (0.16, 1.47)
Allergen sensitized only	635	1.27 (0.85, 1.90)		358	1.078 (0.68, 1.70)
Allergen sensitized and asthma	76	2.53 (1.43, 4.46)	0.011	47	1.55 (0.36, 6.67)	0.210
Hay fever
No sensitization, no hay fever	806	1.00		365	1.00
Hay fever only	19	2.28 (1.12, 4.64)		4	0.03 (0.004, 0.31)
Allergen sensitized only	647	1.37 (0.92, 2.06)		387	1.08 (0.70, 1.65)
Allergen sensitized and hay fever	69	1.87 (1.02, 3.44)	0.310	19	1.57 (0.29, 8.41)	0.011
Rhinitis
No sensitization, no rhinitis	591	1.00		310	1.00
Rhinitis only	235	1.21 (0.87, 1.69)		61	0.61 (0.31, 1.21)
Allergen sensitized only	455	1.40 (0.89, 2.21)		280	0.95 (0.59, 1.55)
Allergen sensitized and rhinitis	260	1.70 (1.07, 2.71)	0.481	126	1.38 (0.63, 3.04)	0.144
Wheeze
No sensitization, no wheeze	730	1.00		351	1.00
Wheeze only	96	1.34 (0.66, 2.73)		20	0.32 (0.10, 1.06)
Allergen sensitized only	594	1.25 (0.84, 1.85)		345	1.05 (0.65, 1.68)
Allergen sensitized and wheeze	123	2.74 (1.55, 4.83)	< 0.001	60	1.57 (0.50, 4.98)	0.047
All models adjusted for age, race, gender, BMI, creatinine, and cotinine. ^***a***^ORs for 1 log_10_ increase in MBzP concentration. ^***b***^*p*-Value for difference of ORs using contrast statement in polytomous model.						

## Discussion

Our most consistent finding was for MBzP and allergic symptoms in adults. MBzP was positively associated with current asthma, current wheeze, current hay fever, and current rhinitis as well as, but nonsignificantly, with allergic sensitization. There was some suggestion that the association between MBzP and allergic symptoms was driven by allergic sensitization, but the evidence was not strong. MBzP is the primary metabolite of BBzP, an HMW phthalate used in plastics and other materials in the home. In a population-based case–control study conducted from 1997 to 2000, Jaakkola et al. (2006) reported that plastic wall materials in the home were associated with incident asthma among 1,453 Finnish adults. There is increasing evidence that MBzP may be associated with allergic outcomes. Prenatal urinary MBzP levels were associated with the development of eczema by 5 years of age in 407 children in a birth cohort study (Just et al. 2012b) and with increased airway inflammation as measured by exhaled nitric oxide (Just et al. 2012a). In a cross-sectional study of 101 Taiwanese children 3–9 years of age, BBzP concentration in house dust was associated with allergic symptoms and asthma (Hsu et al. 2011). Although some phthalates, particularly di-*n*-butyl phthalate, are included in pharmaceuticals (Hernández-Díaz et al. 2009), BBzP is not approved for pharmaceutical use and, thus, MBzP in urine is unlikely to be a consequence of the use of allergy or asthma medications.

Much of the mechanistic work to date on phthalates and allergy has focused on DEHP and its ability to modulate responses to allergens (Jaakkola and Knight 2008; Kimber and Dearman 2010). In murine models and in human lung epithelial cells, DEHP, but not BBzP, has been shown to have an adjuvant effect on immune response to allergens (Guo et al. 2012; Koike et al. 2009; Larsen et al. 2007; Nishioka et al. 2012). In a small human study, 16 adults with sensitivity to house dust mites and 16 without sensitivity were exposed to airborne dust containing low or high levels of DEHP. Those exposed to high levels had an attenuated immune response, whereas those exposed at low levels had mucosal inflammation and nonsensitized individuals had no response (Deutschle et al. 2008). In murine models, BBzP enhanced anti-ovalbumin responses at high doses, but not at the lower doses potentially more consistent with human exposures (Dearman et al. 2009). Few studies have evaluated whether BBzP, or its metabolite MBzP, have independent effects on immune responses at levels relevant to human exposure. One study reported that topical administration of BBzP did not stimulate an immune response in mice (Butala et al. 2004). Our results for DEHP and allergic sensitization in adults are consistent with the mechanistic data; however, we have no information on allergen exposure.

LMW phthalates were not positively associated with allergic symptoms or sensitization, except for MEP and allergic sensitization among Mexican Americans. All other race/ethnicity groups had inverse associations between sIgE and MEP. In addition, MEP was also inversely associated with hay fever and allergic sensitization in adults. In a previous NHANES sample (1999–2000), MEP levels among Mexican Americans were not different from non-Hispanic whites and were lower than for non-Hispanic blacks (Silva et al. 2004), suggesting exposure level did not explain the observed difference. Interestingly, in a study of Dominican (67%) and African-American (33%) children 5–9 years of age, Just et al. (2012a) reported that children with higher urinary levels of MEP had higher fractional exhaled nitric oxide. There was limited evidence for differences between Dominican and African-American children for MEP exposure and allergic sensitization in children, but the sample sizes were small. In a Japanese cross-sectional study of 134 residents of 41 dwellings conducted in 2006–2007, DEP was inversely associated with respiratory and allergic symptoms in both children and adults, consistent with our results (Kanazawa et al. 2010). It is possible that both age and race/ethnicity may influence allergic response to MEP, but currently the data are too limited to explore this extensively. MEP is the primary metabolite of DEP, a phthalate primarily used in fragrances (Api 2001). Some fragrances can be 25–50% DEP by volume (Agency for Toxic Substances and Disease Registry 1995). The inverse association with MEP and sensitization among adults could suggest fragrance avoidance by allergen-sensitized individuals. Among Mexican-American adults, we saw a positive association with MEP. Other investigators have noted that Mexican women who used multiple cosmetic and fragrance products had higher levels of MEP than those who did not, consistent with DEP exposure through the use of fragrances (Romero-Franco et al. 2011). The differential findings for Mexican Americans warrant further characterization of their exposures in the future.

Biological markers of phthalate metabolites are often used to assess exposure because of the complexity of evaluating all sources of exposure. Although the use of biological markers is common, these phthalate measures are limited with respect to the time period they represent. The biological half-lives of these metabolites are < 1 day, and studies have shown that temporal variability in these measures limits their usefulness in estimating the associations with long-term exposure (Baird et al. 2010; Hauser et al. 2004; Hoppin et al. 2002). Given the short biological half-lives of phthalates and the relatively nonvarying state of allergen-specific IgE in serum, our findings for allergic sensitization and HMW phthalates should be considered cautiously. These findings might reflect a preference for plastics among those with allergic sensitization because these surfaces are easier to clean and less likely to be reservoirs for dusts and other allergic triggers. In contrast, our findings for allergic symptoms are less challenged by the use of biological markers because symptoms were experienced within the past year, although daily variation in exposure may influence these findings as well.

Our limited findings for children were unexpected given the previous literature. HMW phthalates, particularly DEHP, were associated with current rhinitis, but none of the associations were statistically significant. In a Swedish study, dust levels of BBzP were associated with rhinitis and eczema in children 3–8 years of age, whereas dust levels of DEHP were associated with asthma; there was also some evidence of an association with rhinitis and DEHP in the highest quartile (Bornehag et al. 2004). Recently, Just et al. (2012b) reported that prenatal exposure to MBzP was associated with maternally reported eczema by 5 years of age and that current exposures were associated with higher fractional exhaled nitric oxide (Just et al. 2012a). The HMW phthalate metabolites, MCOP and MCNP [mono(carboxynonyl) phthalate], were associated with current asthma in a cross-sectional study of 623 Norwegian 10-year-old children (Bertelsen et al. 2013); these two metabolites were not associated with respiratory symptoms in children or adults in our sample. In our analysis, there was no evidence of an association of any phthalates with current itchy rash in either children or adults (data not shown). The lack of consistency with other studies may be related to the age of the children included in our analysis because no children < 6 years of age had data on urinary phthalates. Another possible explanation is that the relevant exposures for children are not those that are currently occurring but rather those that occurred earlier, such as prenatally. In addition, our study included a large representative sample of children in the United States, whereas previous studies used smaller, more demographically similar groups. It may be that the diversity in our sample limited our ability to observe effects restricted to subsets of the population.

Phthalate metabolites, although chemically similar, are not highly correlated except for those derived from the same parent compound. To minimize confounding by correlated phthalates, we combined all metabolites from the same chemical (i.e., DEHP) into one summary variable. Given the multiple phthalates and ways to consider allergy and allergic sensitization, we conducted many statistical analyses. Rather than correcting our results for the number of comparisons or limiting our presentation to those chemicals for which we had *a priori* evidence, we have chosen to present our results uncorrected for multiple comparisons and present the full results in order to provide a more complete picture of the complexity of this research question.

Our previous work has shown poor concordance between self-reported respiratory symptoms and allergic sensitization (Hoppin et al. 2011). To evaluate if our results for respiratory symptoms were driven by allergic sensitization, we ran additional models for those who were both allergen sensitized and had the allergic symptom. These results suggested some interplay of allergic sensitization and symptoms in response to phthalates, but the evidence was inconclusive.

## Conclusions

This study is the largest to date to evaluate associations between phthalates and allergic sensitization and symptoms in both adults and children. Although not entirely consistent with previous studies, our study does provide additional evidence that phthalates, particularly HMW phthalates, may be associated with allergic symptoms in adults and possibly children. The findings were stronger in adults because HMW phthalates were generally positively associated with both allergic sensitization and symptoms in adults, but positively associated only with rhinitis in children. The inverse association estimated for MEP and allergic sensitization is consistent with avoidance of compounds containing DEP by people who are allergen sensitized. We conducted this cross-sectional analysis of urinary phthalate levels and allergic sensitization and symptoms in a large nationally representative racially diverse sample, although generalizability to younger children is limited by the lack of phthalate data for children < 6 years of age. Future studies should not only better characterize the temporal association between exposure and outcome, but they should also include measures such as allergen exposure in order to better understand the potential mechanisms by which phthalates may contribute to allergic outcomes.

## Supplemental Material

(229 KB) PDF
