# Supplementary material for: Phthalate Exposure and Allergy in the U.S. Population: Results from NHANES 2005–2006
Source: Environ Health Perspect. 2013 Jun 25;121(10):1129–34. doi: 10.1289/ehp.1206211 (PMC3801456; doi:10.1289/ehp.1206211)
Supplement: (229 KB) PDF [file ehp.1206211.s001.pdf]

Environmental Health Perspectives doi:10.1289/ehp.1206211

Supplemental Material

Phthalate Exposure and Allergy in the U.S. Population: Results from NHANES 2005-2006

Jane A. Hoppin, Renee Jaramillo, Stephanie J. London, Randi J.Bertelsen, Päivi M. Salo, Dale P. Sandler, and Darryl C. Zeldin

Supplemental Material, Table S1. Urinary Phthalate Concentration (ng/mL) - Survey Weighted Summary Statistics, Children (age 6-17): N=779

	Phthalate	LOD	Pct > LOD	Geo Mean	SE Geo Mean	5th pctl	25th pctl	50th pctl	75th pctl	95th pctl
	Low Molecular Weight			151.18	4.65	26.10	69.59	136.81	303.69	1217.34
MnBP	Mono-n-butyl phthalate	0.6	99.7	29.09	1.35	4.98	15.80	31.56	57.63	134.95
MEP	Mono-ethyl phthalate	0.5	100.0	85.52	4.02	11.56	32.64	71.03	212.76	1050.90
MiBP	Mono-isobutyl pthalate	0.3	99.6	8.15	0.40	1.14	4.07	8.93	16.38	45.97
MMP	Mono-n-methyl phthalate	1.1	46.5	1.83	0.09	< LOD	< LOD	< LOD	3.86	15.74
	High Molecular Weight			175.08	9.10	27.94	87.08	170.63	322.93	1149.91
MBzP	Mono-benzyl phthalate	0.2	99.4	16.82	0.92	2.42	7.69	17.98	37.79	106.75
MCOP	Mono(carboxyoctyl) phthalate	0.7	98.0	7.42	0.49	1.14	3.95	7.23	13.46	49.58
MCNP	Mono(carboxynonyl) phthalate	0.6	94.2	3.75	0.23	< LOD	2.11	3.90	6.98	18.94
MCPP	Mono-(3-carboxypropyl) phthalate	0.2	99.3	3.89	0.27	0.63	2.03	4.09	7.67	17.91
MCHP	Mono-cyclohexyl Phthalate	0.6	2.0	0.44	0.01	< LOD	< LOD	< LOD	< LOD	< LOD
MiNP	Mono-isononyl phthalate	1.2	14.5	1.05	0.03	< LOD	< LOD	< LOD	< LOD	3.16
MOP	Mono-n-octyl phthalate	1.8	1.0	1.32	0.01	< LOD	< LOD	< LOD	< LOD	< LOD
	∑Diethylhexyl phthalate (DEHP)			118.44	7.04	15.36	54.15	114.57	230.02	965.48
MEHP	Mono-(2-ethyl)-hexyl phthalate	1.2	72.2	3.27	0.20	< LOD	< LOD	2.80	6.74	32.73
MECPP	Mono-2-ethyl-5-carboxypentyl phthalate	0.6	100.0	54.81	3.27	7.14	25.29	51.41	101.93	468.39
MEHHP	Mono-(2-ethyl-5-hydroxyhexyl)	0.7	99.9	34.55	2.15	4.10	15.16	33.43	74.70	288.06
MEOHP	Mono-(2-ethyl-5-oxohexyl)	0.7	99.8	22.88	1.38	2.69	10.95	22.50	46.08	185.23

Below Detection Fill Values Included: Fill Value = Lower LOD / sqrt(2) imputed values were used for calulation of geometric mean and SE.

Limited to participants with all covariates in logistic regression model: phthalates, age, race/ethnicity, gender, cotinine, BMI, creatinine

Supplemental Material, Table S2. NHANES 2005-06: Urinary Phthalate Concentrations (ng/mL) - Survey Weighted Summary Statistics, Adults age 18+: N=1546

	Phthalate	LOD	Pct > LOD	Geo Mean	SE Geo Mean	5th pctl	25th pctl	50th pctl	75th pctl	95th pctl
	Low Molecular Weight									
MnBP	Mono-n-butyl phthalate	0.6	99.5	17.99	0.88	2.66	9.42	18.58	36.85	101.08
MEP	Mono-ethyl phthalate	0.5	99.5	114.97	7.79	12.11	40.23	108.36	304.04	1501.59
MiBP	Mono-isobutyl pthalate	0.3	96.6	4.72	0.31	0.39	2.31	5.42	10.53	28.98
MMP	Mono-n-methyl phthalate	1.1	36.2	1.44	0.07	< LOD	< LOD	< LOD	2.24	12.23
	High Molecular Weight									
MBzP	Mono-benzyl phthalate	0.2	98.2	7.08	0.46	0.57	3.18	7.57	17.37	57.37
MCOP	Mono(carboxyoctyl) phthalate	0.7	94.5	5.00	0.36	< LOD	2.14	4.51	9.97	52.79
MCNP	Mono(carboxynonyl) phthalate	0.6	88.9	2.53	0.12	< LOD	1.19	2.41	4.70	17.00
MCPP	Mono-(3-carboxypropyl) phthalate	0.2	95.4	1.78	0.08	0.17	0.87	1.74	3.59	11.26
MCHP	Mono-cyclohexyl Phthalate	0.6	2.2	0.44	0.00	< LOD	< LOD	< LOD	< LOD	< LOD
MiNP	Mono-isononyl phthalate	1.2	12.7	1.05	0.02	< LOD	< LOD	< LOD	< LOD	3.63
MOP	Mono-n-octyl phthalate	1.8	1.1	1.32	0.00	< LOD	< LOD	< LOD	< LOD	< LOD
	∑Diethylhexyl phthalate (DEHP)			79.85	4.00	10.98	33.23	71.01	160.81	918.31
MEHP	Mono-(2-ethyl)-hexyl phthalate	1.2	65.4	2.96	0.13	< LOD	< LOD	2.25	6.19	41.72
MECPP	Mono-2-ethyl-5-carboxypentyl phthalate	0.6	100.0	35.52	1.87	4.69	15.29	31.18	74.83	379.07
MEHHP	Mono-(2-ethyl-5-hydroxyhexyl)	0.7	99.8	23.76	1.18	2.70	9.59	21.40	50.46	311.00
MEOHP	Mono-(2-ethyl-5-oxohexyl)	0.7	98.7	15.01	0.78	1.76	6.10	13.48	33.02	183.53

Below Detection Fill Values Included: Fill Value = Lower LOD / sqrt(2); imputed values were used for calulation of geometric mean and SE.

Limited to participants with all covariates in logistic regression model: phthalates, age, race/ethnicity, gender, cotinine, BMI, creatinine
